# Diffuse reflectance and fluorescence spectroscopy for breast conserving surgery

**DOI:** 10.1007/s10549-025-07790-8

**Published:** 2025-08-01

**Authors:** Dhurka Shanthakumar, Vadzim Chalau, Yufeng Shi, Ria Ranjitkar, Anna Silvanto, Ara Darzi, Daniel R. Leff, Daniel S. Elson

**Affiliations:** 1 Department of Surgery & Cancer, Imperial College London, London, UK; 2 Department of Surgery & Cancer, Imperial College Healthcare NHS Trust, London, UK

**Keywords:** Breast cancer, margins, Intraoperative margin assessment, spectroscopy, Intrinsic fluorescence, Diffuse reflectance spectroscopy

## Abstract

**Purpose:**

The major challenge in breast conserving surgery is the high rates of re-excision due to positive resection margins. This study evaluates whether a combined diffuse reflectance spectroscopy (DRS) and laser induced intrinsic fluorescence spectroscopy (IFS) technique can differentiate breast tissue sample types, towards the development of an intraoperative margin assessment tool.

**Methods:**

Breast tissue samples were collected from patients undergoing breast cancer surgery. A handheld DRS-IFS probe was used on the frozen thawed ex-vivo  breast samples to acquire spectral data. Machine learning classifiers were used to determine sensitivity, specificity, overall diagnostic accuracy, and the area under the curve (AUC) against “gold-standard” histopathology ground truth.

**Results:**

181 breast tissue samples from 138 patients were interrogated using DRS-IFS. All patients were female, with median age (range) of 56.8 (20–94) years The total number of spectra acquired was 18,349. Following five-fold cross validation for normal *versus* cancer tissue, extreme gradient boost classifier achieved a sensitivity of 84% (SD ± 13), specificity of 61% (SD ± 16), overall diagnostic accuracy of 75% (SD ± 3), and AUC of 84%.

**Conclusion:**

The results suggests that DRS-IFS can distinguish normal breast tissue from breast cancer with high diagnostic accuracy. For DRS-IFS to be translated into the operating theatre to aid a surgeon’s real-time visualisation for oncologic margin control assessment of intraoperative, the in vivo diagnostic accuracy needs to be determined.

**Supplementary Information:**

The online version contains supplementary material available at 10.1007/s10549-025-07790-8.

## Introduction

Breast conserving surgery (BCS) is the favoured approach to managing early-stage breast cancer [1]. In BCS, the goal is to excise the lesion with a rim of healthy tissue, while preserving cosmesis. However, on average, 19% (range 9 to 33%) of patients require further surgery due to positive margins [2]. A positive margin refers to pre-invasive or invasive disease at or too close to the cut edge of the specimen. The recent “Getting It Right First Time” report recommends effective intraoperative margin assessment techniques could help reduce re-excision rates [2]. However, current intraoperative techniques such as specimen radiography suffer from poor sensitivity and specificity [3]. Traditional approaches such as frozen section and imprint cytology are highly accurate, but require a pathologist to be present and impedes surgical workflow [4].

Novel intraoperative margin assessment (IMA) technologies are being researched globally to improve precision in BCS. These technologies can be broadly categorised into four groups based on their methodology: imaging-based (X-ray or magnetic resonance imaging (MRI)); optical based; detecting the electrical properties of tissue; and those technologies which exploit the biochemical difference in tissue [5]. Although imaging-based technologies such as CT or MRI have good accuracy in cancer detection, they are expensive to purchase and maintain and require radiological training for interpretation of images [6]. Comparatively, the other groups of technologies described have been investigated and are more user friendly intraoperatively; however, they have not gained widespread usage [7]. We propose that diffuse reflectance spectroscopy combined with intrinsic fluorescence spectroscopy (DRS-IFS) [8] could be utilised for IMA to aid a surgeon’s visualisation to guide tumour excision. DRS-IFS offers an non-invasive, economical IMA method, with a minimal learning curve.

Optical spectroscopy is a technique that harnesses the light-tissue interaction. Light shone onto biological tissue undergoes reflection, refraction, scattering and absorption due to tissue structures and cellular components [9]. In breast tissue, scattering varies in relation to fibroglandular content and collagen, reflective of breast density. Light is absorbed mainly by haemoglobin and lipid [9, 10] although absorption by beta-carotene also occurs in breast tissue [11]. Fluorescence is a further interaction whereby light is absorbed by molecules called fluorophores and re-emitted at a longer “red-shifted” wavelength, leading to differences in emission spectra. Fluorescence spectroscopy can utilise intrinsic fluorophores, or use FDA approved exogenous agents such as indocyanine green or 5-aminolevunilic acid [12, 13]. Our DRS-IFS system exploits intrinsic breast tissues fluorophores, namely nicotinamide adenine dinucleotide (NADH), flavin adenine dinucleotide (FAD), collagen, elastin and lipo-pigments [10]. These fluorophores are associated with cancer progression [14, 15]. These combined processes provide an “optical fingerprint” for tissue characterisation.

Although previous studies have utilised a combination of diffuse reflectance and fluorescence spectroscopy to interrogate breast tissue [16], this study offers several novel aspects. Firstly, a combination of space-resolved DRS at different source-to-detector separations (SDS) and IFS were applied for breast tumour diagnostics along with machine learning algorithms for classification. DRS monopolises on both scattering and absorption characteristics, whereas IFS is sensitive to endogenous fluorophores. The combination of DRS and IFS therefore allows a comprehensive assessment of tissue morphology and composition. Although frozen tissue has been utilised to facilitate specimen slicing [17], freshly frozen breast tissue, as used here, has not been investigated systematically. Optical properties are altered by the freeze–thaw process, and this is a limitation of this work; however, the use of these tissues still provides a rich source of data which can be utilised for clinical translation. A substantially greater number of spectral data have been acquired here compared to previous work [18]. Critically, here, ductal carcinoma insitu (DCIS) has been interrogated, which is important given recent data suggesting DCIS increases the risk of a positive margin more than eight fold [19]. Finally, we include tissue samples from patients who received neoadjuvant chemotherapy (NACT). To date, there is limited research into how optical characteristics of breast tissues change following chemotherapy. It is vital that margin technologies work following NACT since it is increasingly utilised not only to downstage tumours pre-operatively, but also to tailor therapy to improve the overall survival in certain immunophenotypes (e.g. triple negative, HER2 enriched disease) [20].

## Methods

### Study setting

Breast tissue samples were collected following consenting patients undergoing elective breast conserving surgery or mastectomy at either Imperial College London NHS Trust (Charing Cross Campus) and Barts Health NHS Trust. Samples were collected immediately after the patient underwent surgery, and freshly frozen without any use of formalin. The samples were from either the edge of specimens or internal surfaces. Imperial College Tissue Bank ethics (R21032-4A) and Breast Cancer Now ethics (REC ref no. 21EE0072) allowed prospective collection of tissues used in this study. The use of DRS on these tissue samples was approved with an additional ethics application (REC ref no. 08/H0719/37).

### Experimental DRS-IFS setup

**Fig. 1:**
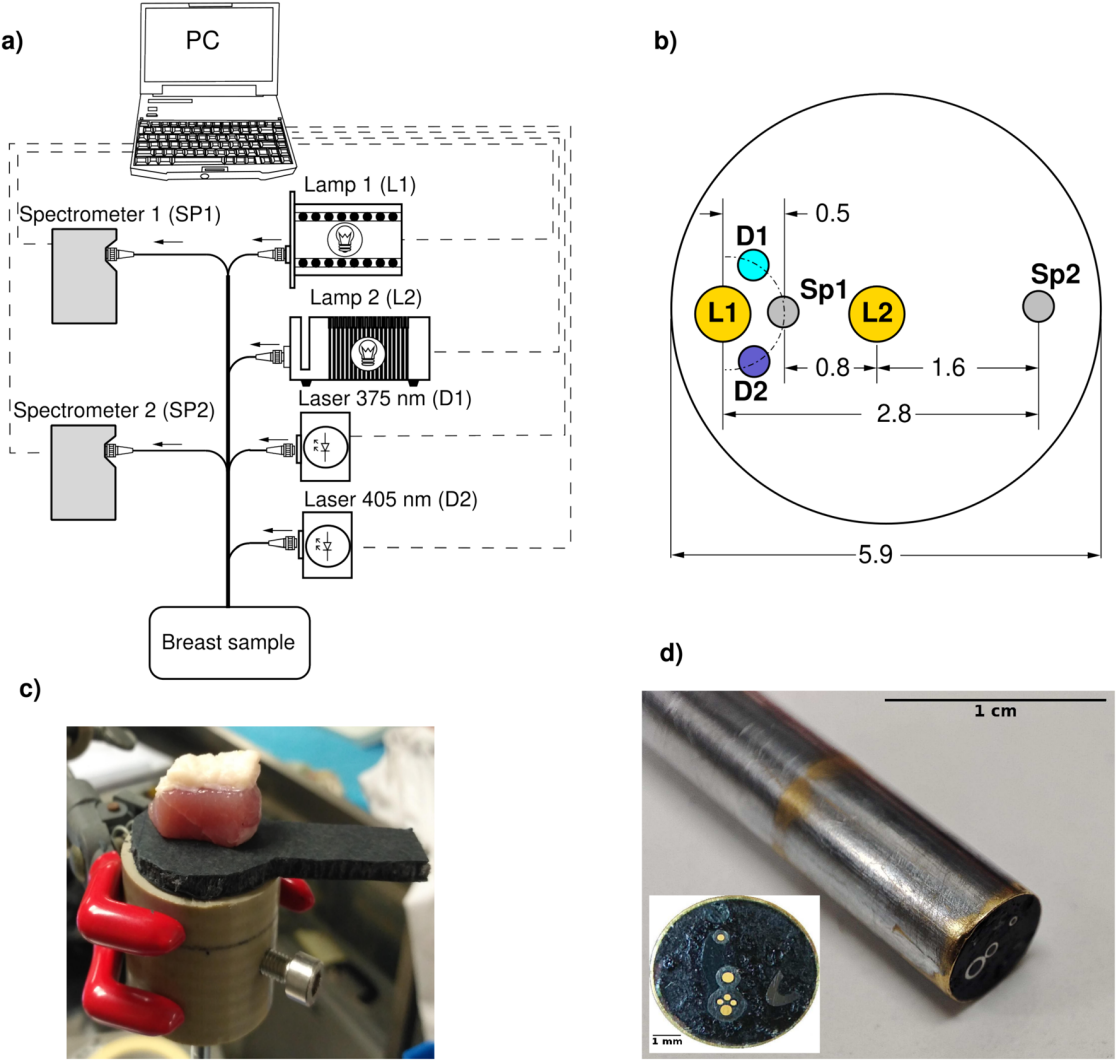
DRS-IFS experimental setup: **a** Schematic illustration depicting the components of the custom-built experimental system. **b** Diagram of configuration of the probe components and distances shown in mm. **c** Sample holder and fibre-optic probe with tissue sample on top and fibre probe in contact with bottom surface of the sample. The spatial resolution of the system is 3–5 mm. The DRS detection volume varies from approximately 0.5–1 mm^3^ at a 0.5 mm fibre separation to several mm^3^ at 2.8 mm fibre separation, and interrogation depth varies from 0.1 mm up to 3.5 mm, although this will be affected by the specific optical properties of each breast tissue sample. For IFS, the detection volume is in order of 1 mm^2^, the interrogation depth is less than 0.5 mm. **d** Photograph of the DRS-IFS multifibre probe distal tip, inset – photograph of the fibre configuration

The experimental setup consisted of broadband light sources, lasers, spectrometers and fibre-optic probe (Fig. 1a) A custom-built six-channel fibreoptic probe (Fig. 1a, b) was used for data acquisition. For DRS mode, two fibres with core diameter 400 μm, connected to broadband tungsten lamps (HL-2000-HP-232R and HL-2000-FHSA-LL, Ocean Optics Inc., USA), were used for illumination and two fibres with core diameter 200 μm were connected to CCD-based compact spectrometers (USB4000-UV-NIR and Flame-S-VIS–NIR, Ocean Optics Inc.,USA) for diffuse reflectance collection. The applied configuration (Fig. 1b) allowed diffuse reflectance spectra to be detected at fibre-to-fibre separations of 0.5, 0.8, 1.6 and 2.8 mm in the spectral range of 425–850 nm. For fluorescence mode, the two fibres with core diameter 200 μm, connected to laser diodes with emission wavelength 375 nm (NDU1113E, Nichia Inc., Anan, Japan) and 405 nm DLS5146-101SP, Thorlabs Inc., USA) used to deliver excitation light to the tissue. Fluorescence spectra in the range 425–850 nm were recorded via a USB4000-UV-NIR spectrometer with installed excitation rejection long pass filter (cut-off wavelength 425 nm, OD = 4, Techspec (R) High Performance filter, Edmund Optics Ltd.).

**Fig. 2:**
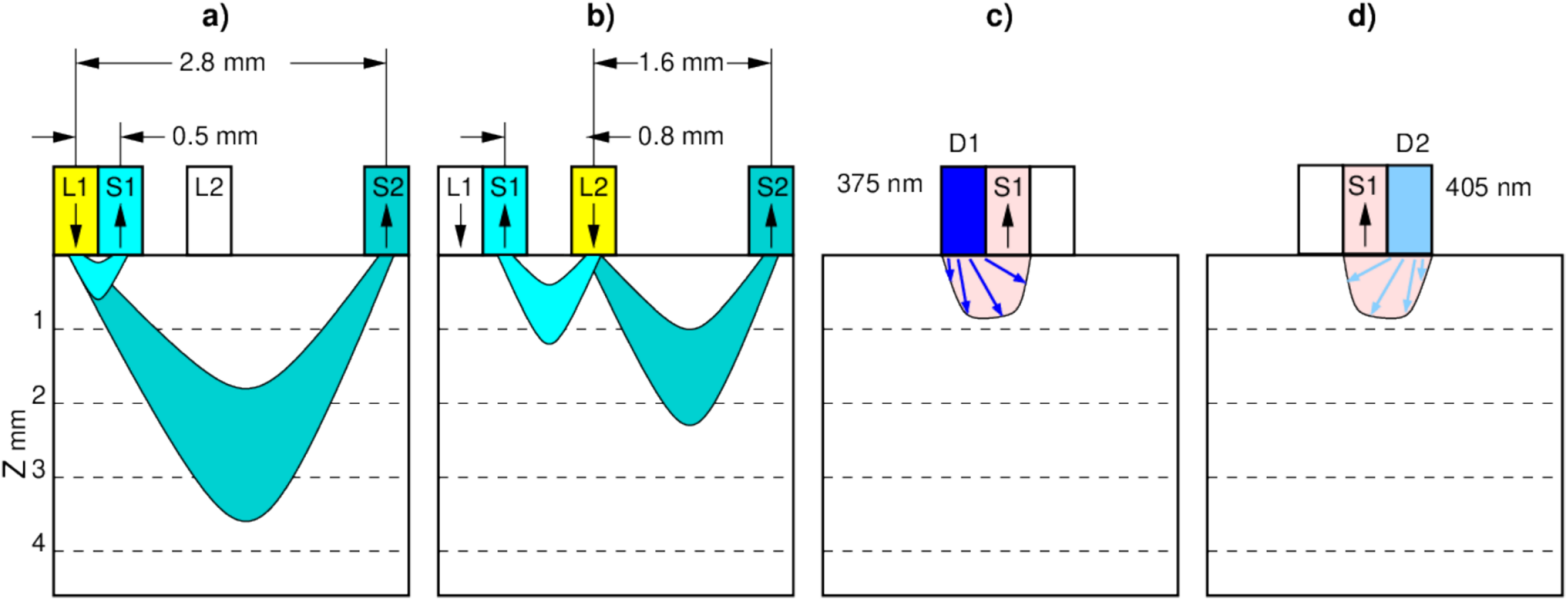
Illustration of the four different acquisition modes of the DRS-IFS system representing DRS and IFS data collection. DRS **a**) and **b**) sampling volumes are approximately represented by the coloured areas, and were estimated via a cloud-based Monte Carlo simulation tool [21] for human fatty breast tissue with absorption coefficient 0.041 cm^−1^ and reduced scattering coefficient 8.5 cm^−1^ for DRS at 786 nm[22]. **a** demonstrates simultaneous acquisition of DRS spectra at distances 0.5 and 2.8 mm, **b** demonstrates simultaneous acquisition of DRS spectra at distances 0.8 mm and 1.6 mm, **c** and **d** demonstrate acquisition of IFS spectra at 375 nm and 405 nm excitation. The tissue depth is depicted by “Z” on the left-hand side and represented in millimetres

By combining two light sources and two spectrometers, within 10–15 s, four DRS and two IFS spectra were recorded. This novel custom-built system allows measurements to be made at multiple source-to-detector fibre separations, as well as including a fluorescence capability within a single acquisition (Fig. 2).

### Data acquisition protocol

Data acquisition occurred within 30 min of the frozen breast tissue samples being thawed to room temperature. The DRS-IFS setup was calibrated in advance to allow for an efficient workflow. A Spectralon® white diffuse reflectance standard (Edmund Optics Ltd, UK) was used for DRS calibration, and a custom-built white fluorescent film for IFS calibration. The acquisition team were blinded to pre-operative histology of each tissue sample to avoid bias.

Each tissue sample was placed on top of the multifibre probe (Fig. 1c) and covered with a black cylindrical cover to obscure ambient light. When the first lamp (L1, Fig. 2a) was enabled, the spectrometers simultaneously detected two DRS spectra at distances 0.5 mm and 2.8 mm (DRS05 and DRS28 further in the text). When the second lamp (L2, Fig. 2b) was enabled, the spectrometers simultaneously recorded two DRS spectra at distances 0.8 mm and 1.6 mm (DRS08 and DRS16). The corresponding IFS spectra were recorded when the 375 nm or 405 nm lasers were on (Fig. 2c and 2 d, IFS375 and IFS405). Typically, the duration of acquisition of the set of six spectra was 10–15 seconds.

Firstly, data were acquired from the anterior surface of the sample, which was rotated over the probe to capture up to 12 spectra at each of 8–10 rotation positions. This process was repeated after turning the sample over to the posterior aspect. On average, 96–120 spectra per tissue sample were acquired giving 18,439 spectra overall from 181 samples across 138 patients.

### Histopathology correlation

**Fig. 3:**
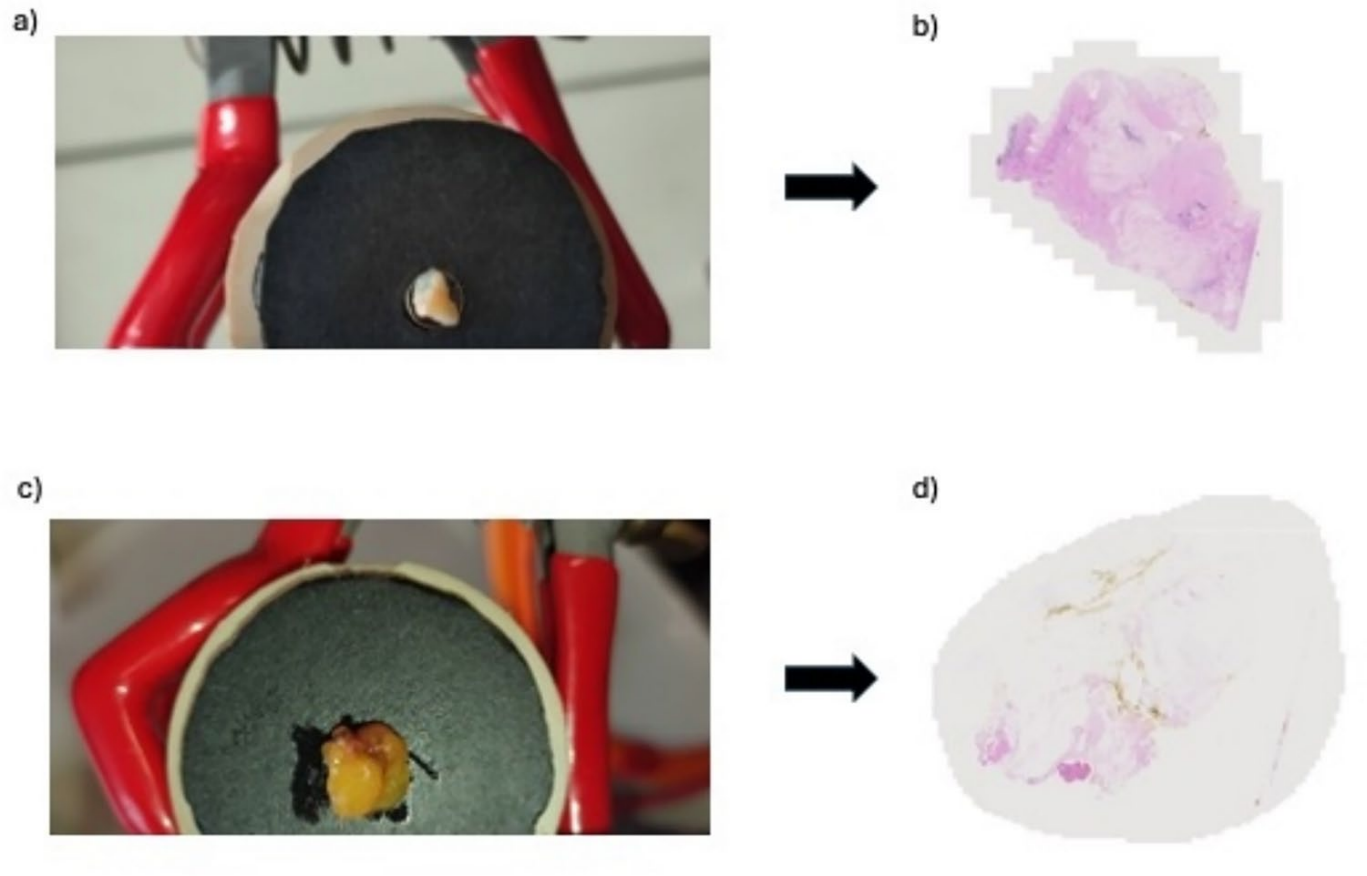
**a** Macroscopic photograph of breast tissue sample interrogated with the DRS-IFS system. **b** Corresponding histopathology slide image following review by an experienced consultant pathologist identified invasive lobular cancer. **c** Macroscopic photograph of a normal breast tissue sample with healthy appearance. **d** Corresponding histopathology slide identifying healthy breast tissue

Two approaches were used for histopathological cross validation. For 122 of the samples, the posterior aspect of the tissue sample was inked with a small yellow dot using tissue dye following data acquisition (Cancer Diagnostics Inc., Durham, NC, USA). The tissue samples were sent to histopathology in formalin and then underwent routine haematoxylin and eosin (H&E) processing. Both aspects of each sample were accounted for in this processing to correlate with data acquisition (yellow dot depicted the posterior side). Macroscopic photographs of the tissue samples (Fig. 3) were also taken. An experienced consultant pathologist, who was blinded, interpreted the H&E slides. The pathologist provided the following data fields: tumour size, histological subtype(s), tumour grade, receptor status, and margin status.

The remaining 59 samples underwent frozen section processing prior to data acquisition. Each sample had several slices taken (~ 4 µm in thickness), with slides read by an experienced consultant histopathologist. Samples were only utilised for data acquisition if it was felt the whole sample represented the tissue type.

### Sample quality assessment

The size and shape of samples varied from 3 × 4 × 2 mm to 27 × 11 × 10 mm, and the ability to collect accurate spectral measurements on the smallest sample was limited due to the typical optical sampling volumes for this specific fibre probe. To assess the influence of this on the overall results, each tissue sample was assigned a quality grading on scale from one (“good quality”) to three (“poor quality”) depending on size dimensions and the presence of blue dye staining (Table 1).

**Table 1:** Summary of number and percentage of samples following quality assessment

Quality score	Criteria for quality	Number of samples	%
1—Good	> 4 mm in size; homogenous; absence of histological or blue dye	106	58.56
2—Mediocre	3 to 4 mm in size; histological or blue dye was present but an area of tissue free of dye was present	47	25.97
3—Poor	< 3 mm in size; heavily contaminated with dye	28	15.47

Samples are categorised at a worse quality if they meet any of the conditions of that category

### Data preprocessing

**Fig. 4:**
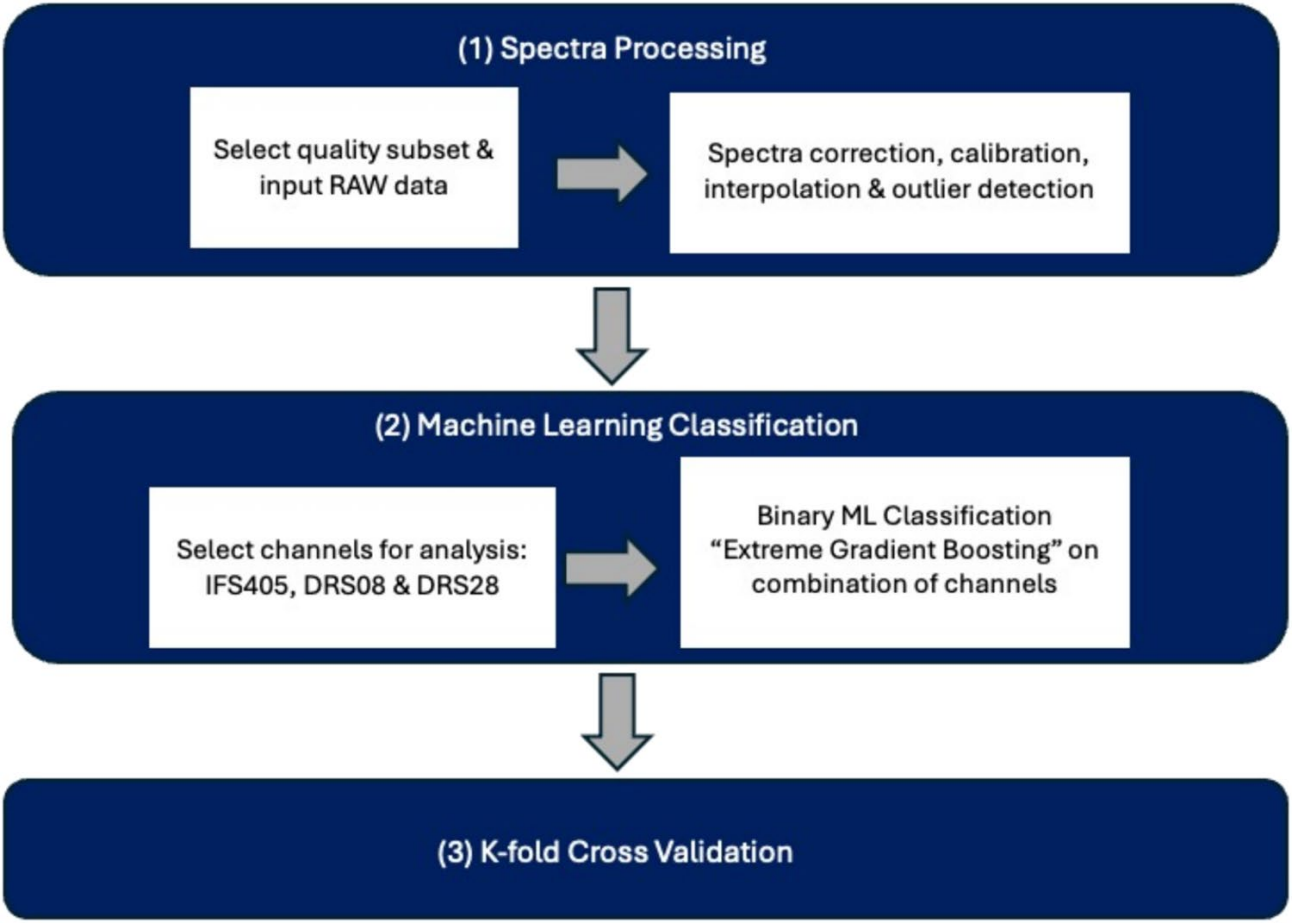
Summary of the main steps involved in preprocessing and classification of the acquired spectra. The classification was repeated for different combinations of the acquired spectra

Preprocessing of spectral data was performed to account for inter-sample, background light, and signal quality variability (Fig. 4). DRS spectra intensities were corrected according to the spectrometer integration time. Spectral shapes were corrected using white standard calibration data and the fluorescence reference data to account for experimental variability. Only spectra with signal-to-noise ratio equal or greater than 5 were included in the analysis. A Savitzky-Golay filter was used to smooth the data and remove noise, whereupon denoised spectra were resampled in the range of 425–850 nm with constant step 1 nm using spline interpolation. Data preprocessing was performed in MATLAB version 9.13 (R2022b) (The MathWorks Inc, 2022).

To reduce the data dimensionality, spectra in the range of 450–850 nm were divided into 20 windows with 20 nm fixed width and 5 spectral features (window mean intensity, window maximal and minimal intensity, and wavelength corresponding to minimum and maximum intensities) were extracted. The Boruta algorithm [23] then extracted a total of 100 features per single spectrum (termed ‘Fixed Windows Features’), which were used as the input for the machine learning classifier.

### Classification

The six-channel probe acquired six different types of spectra: four channels for DRS (DRS05, DRS08, DRS16, and DRS28 for separations 0.5, 0.8, 1.6, and 2.8 mm, respectively) and two channels for IFS (FL375 and FL405 for fluorescence spectra, excited at 375 nm and 405 nm, respectively). Spectra were labelled using tissue subtype categories to perform different comparisons, e.g. healthy vs invasive ductal cancer (IDC); healthy vs invasive lobular cancer (ILC); healthy vs ductal carcinoma in situ (DCIS); healthy vs pathological complete response (PCR); healthy vs invasive cancer (IDC and ILC); healthy vs all cancer (IDC and ILC and DCIS). A tree-based classifier, extreme gradient boosting (XGB) was used for all the described comparisons and for all spectral features, and different combinations of individual channels (Supplementary materials). Software was developed in Python 3.10 for model training, tumour classification, and model evaluation using the Scikit-learn 1.1.3 [24] and XGBoost 1.7 toolkits [24, 25].

The dataset was divided into a training and a testing set using the repeated stratified *k*-fold cross-validation method by patient, with five folds and five repeats. The training dataset was used during the training process while the testing dataset was only used for evaluating the model.

## Results

**Fig. 5:**
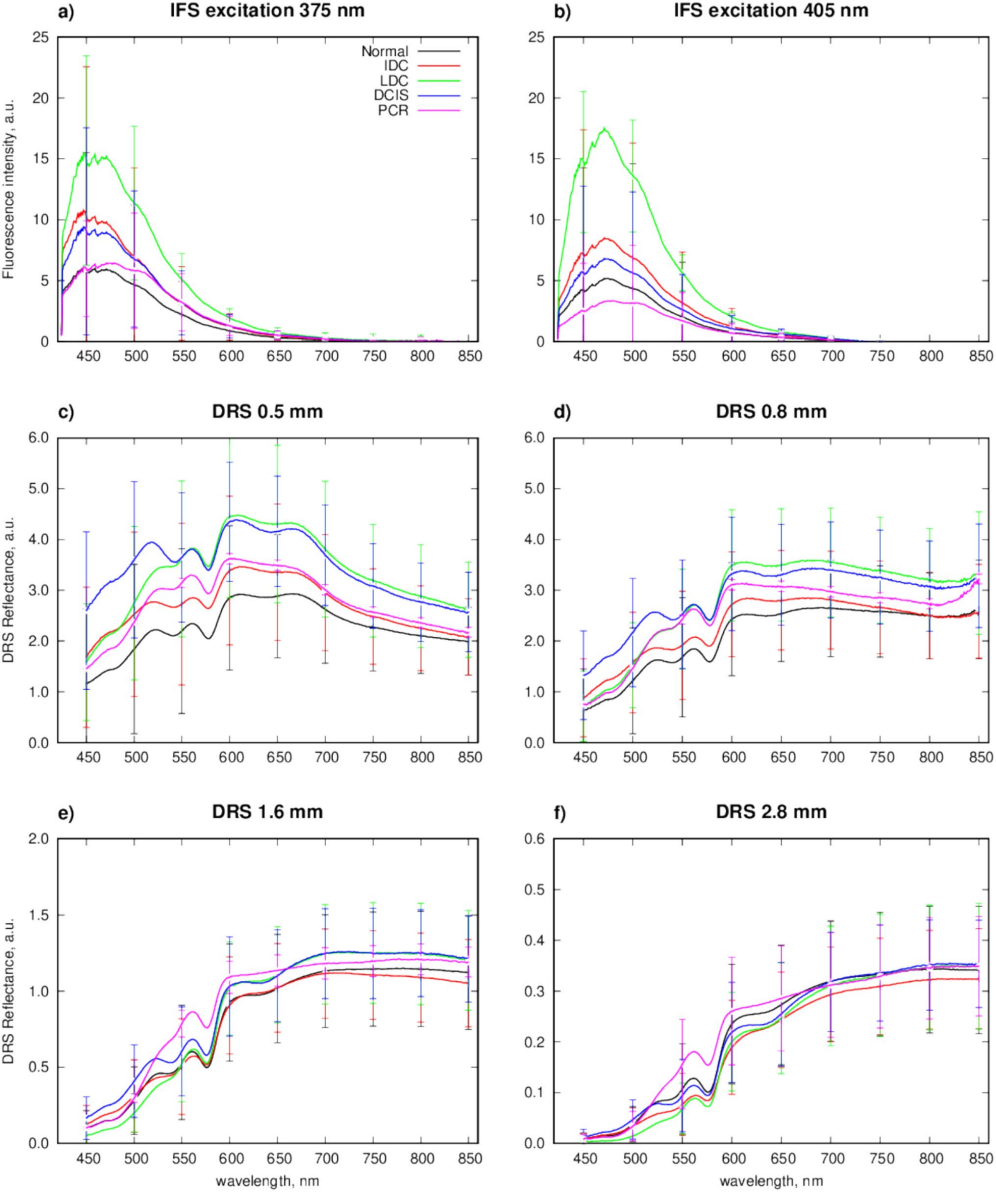
Graphs depicting mean spectra and standard deviations (represented by vertical bars) from each individual channel after preprocessing of data from all tissue samples regardless of quality: fluorescence excitation at (**a**) 375 nm, (**b**) 405 nm (**c**–**f**) spectral features from DRS at (**c**) 0.5 mm, (**d**) 0.8 mm, (**e**) 1.6 mm, (**f**) 2.8 mm. *DRS* diffuse reflectance spectroscopy, *IFS* intrinsic fluorescence spectroscopy

Table 2 summarises patient demographics and histopathological data including tumour subtype, nuclear grade, and receptor status, as well as the number of spectra acquired. Figure 5 depicts the mean spectrum for each individual channel after processing.

**Table 2:** Tissue samples categorised into histopathological subtype; tumour grade and receptor status for all tissue samples, regardless of quality, interrogated with the custom-built probe

Tissue subtype	Total number of samples	Mean age (Range)	Grade	Receptor status
IDC	46	57.3 (34–91)	1: 1 2: 28 3: 17	ER + PR + HER2−: 29 ER + PR−HER2−: 2 Triple −ve: 15
ILC	46	65.7 (43–94)	1: 0 2: 35 3: 11	ER + PR + HER2−: 42 ER + PR−HER2−: 4 Triple −ve: 0
DCIS	21	51.8 (25–76)	Low: 1 Intermediate/high: 11 High: 9	Not applicable
Healthy	62	56.7 (20–90)	Not applicable	ER + PR + HER2−: 55 ER + PR−HER2−: 2 Triple −ve: 4 Unknown: 11
PCR	6	54.8 (40–88)	Not applicable	ER + PR + HER2−: 2 ER + PR−HER2−: 0 Triple −ve: 4

Tissue subtype abbreviations: *IDC* invasive ductal carcinoma, *ILC* invasive lobular carcinoma, *DCIS* ductal carcinoma in-situ, *PCR* pathological complete response to neoadjuvant chemotherapy; Receptor status abbreviations: *ER* +  oestrogen receptor positive, *PR* +  progesterone receptor positive, *PR*− progesterone receptor negative; HER2−- = Herceptin receptor negative; Triple −ve = oestrogen, progesterone, and Herceptin receptors are all negative. The amount of spectral data collected is also tabulated

Following initial assessment, the best results were identified to be from Fixed Windows Features, from a combination of IFS405, DRS08, and DRS28 channels, following validation with XGB classifier. Therefore, the results are provided for this combination of spectral features and channels.

Table 3 presents the normal vs malignant breast tissue (invasive and DCIS) classification statistics and accounts for the sample quality for the combination of DRS 0.8 mm, DRS 0.28 mm, and IFS 405 nm channels. Quality one samples had marginally better results for specificity and AUC, but otherwise, the quality of samples did not affect measurements significantly. The DRS-IFS probe was able to differentiate between normal and malignant tissue with an accuracy of 75.2% (SD ± 3), a sensitivity of 83.5% (SD ± 13), and specificity of 61.1% (SD ± 16), with the XBG classifier on the best quality samples.

**Table 3:** Comparisons between healthy and malignant breast tissue samples (IDC & ILC & DCIS) including accuracy, sensitivity, specificity, area under the curve (AUC), and mean average precision (mAP), using ‘fixed window’ features from a combination of channels DRS 0.8 mm, DRS 2.8 mm, and IFS 405 nm

Sample quality	Accuracy (%)	Sensitivity (%)	Specificity (%)	AUC (%)	mAP (%)
All (1 to 3)	76.1 ± 3	85.6 ± 2	56.7 ± 9	81.3 ± 4	89.3 ± 4
1 “Good”	75.2 ± 3	83.5 ± 13	61.1 ± 16	84 ± 3	89 ± 3

The comparisons are made for either all tissue samples (despite their quality score) or only the best quality tissue samples (quality score of 1) using the XGB classifier.

Accuracy rates for detection of healthy tissue and between different histological subtypes were compared (Table 3 to 6 within Supplementary Materials). DRS-IFS was able to differentiate between healthy and ILC (accuracy of 80.2% (SD ± 14), sensitivity of 73.3% (SD ± 20), and specificity of 86.1% (SD ± 87). The accuracy of IDC was inferior to ILC detection (accuracy of 72.0% (SD ± 11), sensitivity of 61.1% (SD ± 18), and specificity of 78.7% (SD ± 17)).

Unique to this study, we interrogated 21 DCIS samples, which accumulated 2520 spectra. The accuracy of the probe to distinguish healthy tissue from DCIS in the “best quality” samples was 83.5% (SD ± 5), with a sensitivity of 67.6% (SD ± 18) and specificity of 90.5% (SD ± 5).

Patients receiving NACT can have a complete pathological response (pCR), where no invasive cancer is seen. Here, pCR was considered as a separate tissue class. From the six samples used for analysis, five yielded good quality and led to an accuracy of 90.2% (SD ± 4), with sensitivity of 35.3% (SD ± 44), and specificity of 97.5% (SD ± 2) when compared to healthy tissue.

The accuracy of distinguishing between tumour classes was much lower for both classifiers regardless of tissue sample quality. IDC and ILC were evenly represented, and the accuracy of discrimination was 62% (SD ± 10).

**Fig. 6:**
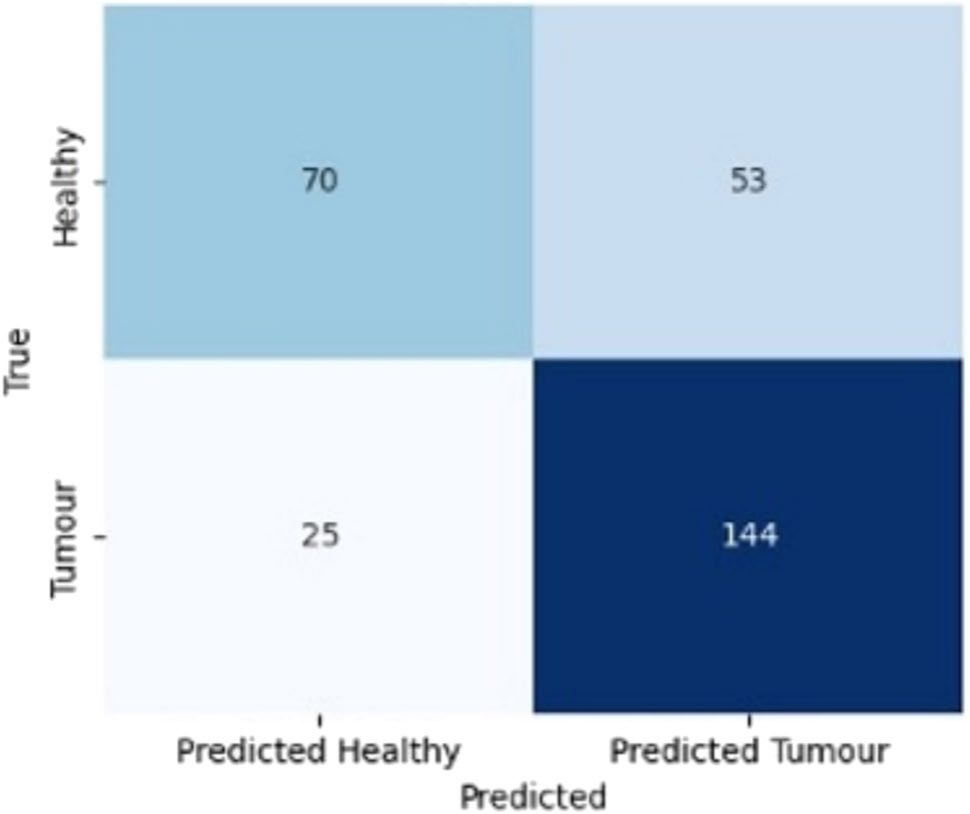
Confusion matrix comparing healthy tissue samples versus all quality one (good) tumour samples (IDC & ILC & DCIS) for the channel combination of DRS 0.8 mm, DRS 2.8 mm, and IFS 405 nm, once the XGB classifier is applied

**Fig. 7:**
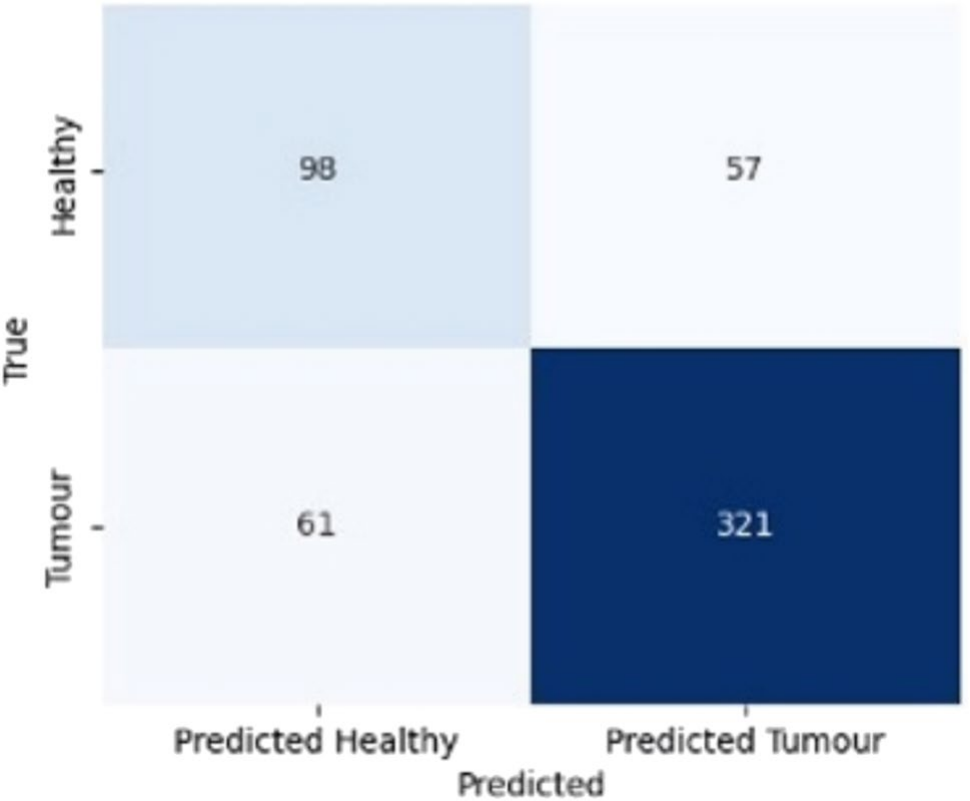
Confusion matrix comparing healthy tissue samples versus all tumour samples (IDC & ILC & DCIS) irrespective of quality score for the channel combination of DRS 0.8 mm, DRS 2.8 mm, and IFS 405 nm and Fixed Windows Features

When healthy tissue samples were compared to all malignant tissue samples (IDC, ILC and DCIS), it is noted that XGB classified the majority of spectra within the ‘predicted tumour’ box (Figs. 6, 7).

## Discussion

Experimental results demonstrate that the combination of DRS and IFS can distinguish between ex-vivo normal and malignant breast tissues when machine learning algorithms are applied. Overall, diagnostic accuracy was comparable to previous spectroscopy studies [26–28], but specificity is notably lower. Possible explanations for inferior specificity are discussed below.

Previous optical spectroscopy diagnostics work has mainly used fresh tissue samples. Our results, obtained with thawed frozen breast tissue samples is a clear limitation of this study. However, it has been shown that freshly frozen samples mirror findings observed in assessment of fresh tissues with other spectroscopy studies as echoed in our recently published meta-analysis [18]. There is no documented literature on how spectral measurements in human breast tissue are affected by freezing and thawing. However, studies investigating various tissue types in animal models have demonstrated optical properties are altered. Ice crystal formation during freezing creates mechanical stress on cell structures pertaining to cell damage [29]. This leads to a 50% decrease in absorption and 30% reduction in scattering coefficients, independent of light wavelength [30], as seen in rat brain and kidney tissues. Similarly, in hamster cheek pouch, there were deviations in diffuse reflectance and intrinsic fluorescence [31]. A decrease in absorption can be attributed to the fact that during the storage process, samples have no access to air and deoxygenation occurs [32]. The freeze/thaw process causes haemoglobin reoxygenation that is released from haemolysed erythrocytes and partial cell membrane damage [33, 34]. Diffuse reflectance is lower following the freeze/thaw process as cell lysis causes more blood to be on the surface of the sample, which increases the effects of haemoglobin absorption [31]. Due to limitations at our research facility whereby the pathology department has a policy whereby freshly excised specimens cannot be cut intraoperatively in order to avoid disruption to histological workflow, we were limited to utilising freshly frozen samples. In the future, any further work should aim to interrogate tissue samples once it has been freshly excised from the patient to negate the effects of freezing.

Methodologically, previous studies have acquired spectra from specific locations on tissue samples, whereby a pathologist has macroscopically identified tissue subtypes, on sliced lumpectomy specimens [17, 27, 35, 36]. This may introduce bias and could account for improved specificity values. *de Boer* et al. used frozen samples, achieving 100% sensitivity and specificity [37]. In our study, blocks were created from tissue samples to assess both the anterior and posterior sides. On some occasions, it is noticed that the tissue sample comprised a heterogeneous mix of tissue types, a possible contributory factor to lower overall accuracy. Thus, the overall tissue class for each specimen was chosen for machine learning.

An imbalance in sample size between histological subtypes may lead to classification performance being biased towards the group of smaller or larger sample size [38]. The supplementary materials highlight confusion matrices which show that where a tissue class had more samples, the XBG classifier showed better accuracy in this class. For example, there was good accuracy in discriminating healthy tissue from pCR or DCIS, but sensitivity was poor. *de Boer* et al. have also demonstrated that DRS can accurately identify DCIS, but noticed that the optical characteristics of IDC and DCIS were quite similar [17]. Future work must employ the strategies to overcome imbalanced datasets such as resampling techniques at the data level to either increase the number of samples in the minority class, or decrease the number of samples in the majority class, or assign different weights to classes at the algorithm level [39, 40].

The current study has several novel aspects, including broad representation of breast tissues, assessment of tissue heterogeneity, and evaluation of tissue sample quality. Lobular cancer and DCIS are known predictors of positive resection margins [41–43], and it is thus imperative to interrogate them with DRS-IFS. Here, DRS-IFS distinguished healthy breast tissue from ILC better than IDC. Morphological differences between lobular and ductal cancer may explain this finding as lobular cancer is composed of small, discohesive cells which infiltrate the stroma in a single-file manner [44]. Here, 46 lobular breast tissue samples were interrogated the largest number to date, as our recent meta-analysis identified spectral data from 14 lobular cancers only [16, 27, 36, 45, 46].

This work has interrogated the largest set of DCIS samples with DRS-IFS with high specificity but lower sensitivity, likely due to imbalanced datasets. DRS-IFS distinguished IDC and DCIS with reasonable accuracy, but poor specificity. From the mean spectra (Fig. 5), the spectral fingerprint for IDC and DCIS appears to be quite similar, which is commensurate with Kho *et al*. who also observed these spectral similarities [17]. Clinically, despite similar features, when IDC or DCIS are detected at a resection margin in-vivo would require further resection. NACT alters tissue structure [47], so an ideal IMA tool should distinguish this but also be able to identify the residual disease or DCIS among tissue.

Sample quality has been critically analysed here, with 16% of samples having significant dye contamination and 40% partial contamination. Spectral measurements are affected by the presence of pigments (surgical patent V or pathological dye) therefore a limitation of optical techniques [48]. Previous studies have failed to address how dye in samples was assessed and/or how dye impacted the results [17, 36, 48]. Bydlon *et al*. assessed the effects of patent blue dye on optical absorption and scattering in the breast [49, 50] and identified dye up to a particular concentration (80 µm) does not impact the extractions of total haemoglobin; ß carotene, and reduced scattering coefficient spectrum from DRS. In the context of modern day breast surgery, any future IMA tool must consider the effects of emerging tracers used for sentinel lymph node biopsy such as indocyanine green and supermagnetic iron oxide which may affect spectral readings. Here, an interesting finding is that the quality of the samples does not statistically significantly affect the diagnostic results. We believe that either this is the result of automatic exclusion of bad spectra from the analysis at the preprocessing stage, or the machine learning-based method ignores spectral artefacts. This issue requires further analysis and study.

## Conclusion

In this study, we present, for the first time, the use of a combined approach employing spatially resolved diffuse reflectance and intrinsic fluorescence spectroscopy for the classification of freshly frozen breast tissue samples, leveraging machine learning algorithms. The achieved sensitivity of 84% (SD ± 13.1), specificity of 61% (SD ± 16.4), overall diagnostic accuracy of 75% (SD ± 3.2), and AUC of 84% are comparable to diagnostic performance observed with freshly excised tissue samples or in-vivo applications. Our results look promising towards the application of machine learning classification with fluorescence and diffuse reflectance spectroscopy to address the challenge of positive margin detection, as well as to expedite the diagnosis and accelerate the acquisition of supplementary investigations. Further research is needed to explore the applicability of the developed machine learning approach to freshly excised and in-vivo tissue samples.

## Supplementary Information

Below is the link to the electronic supplementary material.

Supplementary file1 (DOCX 218 KB)

## Data Availability

The raw data (DRS and IFS spectra) and trained machine learning training data are not publicly available (potential IP protection) but are available from the corresponding author on reasonable request.

## Abbreviations

BCS: Breast conserving surgery
DRS: Diffuse reflectance spectroscopy
IFS: Intrinsic fluorescence spectroscopy
IMA: Intraoperative margin assessment
NADH: Nicotinamide adenine dinucleotide
FAD: Flavin adenine dinucleotide
DCIS: Ductal carcinoma in situ
NACT: Neoadjuvant chemotherapy
SDS: Source-to-detector separations
XGB: Extreme gradient boosting
pCR: Pathological complete response
VAB: Vacuum-assisted biopsy
