# Supplementary material for: Diffuse reflectance and fluorescence spectroscopy for breast conserving surgery
Source: Breast Cancer Res Treat. 2025 Aug 1;214(1):25–36. doi: 10.1007/s10549-025-07790-8 (PMC12398437; doi:10.1007/s10549-025-07790-8)
Supplement: Supplementary file 1 — Supplementary file1 (DOCX 218 KB) [file 10549_2025_7790_MOESM1_ESM.docx]

**Supplementary Materials**

**Table 1.** AUC for individual channels and for different combinations of channels – all tissue samples. Here ‘D’ represents diffuse reflectance spectroscopy, and ‘f’ is fluorescence spectroscopy. The numbers in the top row adjacent to ‘D’ are related to the distance from the lamp to spectrometer (e.g. D8 = 0.8 mm from lamp two and spectrometer one as depicted in Figure 1); and ‘f3’ means laser diode at 375 nm and ‘f4’ is where laser diode at 405 nm.

| **Type of Exp** | **D8** | **D28** | **f3** | **f4** | **D8_f3** | **D8_f4** | **D28_f3** | **D28_f4** | **D8_28_f3** | **D8_28_f4** | **D8_f3_4** | **D28_f3_4** | **D8_28_f3_4** |
| --- | --- | --- | --- | --- | --- | --- | --- | --- | --- | --- | --- | --- | --- |
| Healthy vs. IDC | **74.85%** | 71.90% | 70.16% | 61.50% | 75.62% | 75.72% | 73.14% | 71.18% | **76.70%** | 74.75% | 76.10% | 71.66% | 76.27% |
| Healthy vs. ILC | 80.68% | **84.36%** | 73.06% | 74.05% | 79.72% | 79.51% | 82.90% | 83.87% | **82.30%** | 81.78% | 78.01% | 81.67% | 82.11% |
| Healthy vs. DCIS | **91.21%** | 83.98% | 77.39% | 80.09% | 88.03% | 89.68% | 84.11% | 84.79% | **87.68%** | 88.14% | 89.97% | 84.47% | 87.94% |
| Healthy vs. PCR | **78.02%** | 60.95% | 53.76% | 50.37% | 82.49% | 79.68% | 63.95% | 54.67% | **82.29%** | 74.93% | 81.18% | 55.77% | 82.73% |
| Healthy vs. IDC+ILC | 77.30% | **77.64%** | 71.63% | 67.67% | 76.00% | 77.40% | 76.94% | 76.14% | **80.59%** | 79.79% | 77.74% | 78.68% | 79.63% |
| Healthy vs. IDC+ILC+DCIS | **79.95%** | 79.74% | 71.75% | 69.72% | 78.93% | 81.67% | 78.53% | 78.13% | **81.01%** | 81.44% | 81.21% | 78.33% | 80.95% |
| Mean AUC - All experiments | **80.33%** | 76.43% | 69.62% | 67.23% | 80.13% | 80.61% | 76.59% | 74.80% | **81.76%** | 80.14% | 80.70% | 75.10% | 81.61% |

**Table 2.** AUC of combining different channels– quality 1 tissue samples. Here ‘D’ represents diffuse reflectance spectroscopy, and ‘f’ is fluorescence spectroscopy. The numbers in the top row adjacent to ‘D’ are related to the distance from the lamp to spectrometer (e.g. D8 = 0.8 mm from lamp two and spectrometer one as depicted in Figure 1); and ‘f3’ means laser diode at 375 nm and ‘f4’ is where laser diode at 405 nm.

| **Type of Exp** | **D8** | **D28** | **f3** | **f4** | **D8_f3** | **D8_f4** | **D28_f3** | **D28_f4** | **D8_28_f3** | **D8_28_f4** | **D8_f3_4** | **D28_f3_4** | **D8_28_f3_4** |
| --- | --- | --- | --- | --- | --- | --- | --- | --- | --- | --- | --- | --- | --- |
| Healthy vs. IDC | 69.59% | **73.24%** | 67.31% | 59.52% | 69.95% | 70.06% | 74.27% | 75.43% | 74.50% | **77.41%** | 71.08% | 77.52% | 77.35% |
| Healthy vs. ILC | 80.94% | **90.86%** | 74.83% | 76.22% | 80.89% | 83.94% | **88.48%** | 86.05% | 86.99% | **86.53%** | 83.68% | 85.98% | 83.84% |
| Healthy vs. DCIS | **81.53%** | 77.07% | 80.49% | 76.20% | 82.42% | **86.78%** | 77.35% | 77.44% | 82.85% | **84.29%** | 88.83% | 74.94% | 85.73% |
| Healthy vs. PCR | **89.41%** | 55.45% | 58.20% | 54.00% | 88.68% | **92.18%** | 61.46% | 61.84% | 84.32% | **87.32%** | 86.98% | 56.99% | 84.67% |
| Healthy vs. IDC+ILC | 77.12% | **80.18%** | 70.51% | 68.56% | 77.51% | 77.61% | 82.57% | 80.13% | 82.94% | **82.42%** | 78.01% | 78.02% | 82.81% |
| Healthy vs. IDC+ILC+DCIS | 77.08% | **79.33%** | 72.02% | 67.18% | 75.99% | 78.96% | 80.16% | 80.27% | 81.33% | **82.93%** | 79.21% | 79.37% | 83.93% |
| Mean AUC - all experiments | **79.28%** | 76.02% | 70.56% | 66.95% | 79.24% | 81.59% | 77.38% | 76.86% | 82.15% | **83.48%** | 81.30% | 75.47% | 83.06% |

**Table 3.** Table depicting AUC (area under the curve) results from individual channel analysis. Here ‘DRS” represents diffuse reflectance spectroscopy, and ‘fl’ represents fluorescence spectroscopy. The numbers in the top row adjacent to ‘DRS’ are related to the distance from the lamp to spectrometer (e.g. D8 = 0.8 mm from lamp two and spectrometer one as depicted in Figure 1); and ‘fl375’ means laser diode at 375 nm and ‘fl405’ is where laser diode at 405 nm.

| **Type of Exp** | **DRS05** | **DRS08** | **DRS16** | **DRS28** | **fl375** | **fl405** |
| --- | --- | --- | --- | --- | --- | --- |
| Healthy vs. IDC | 67.88% | 68.77% | 70.16% | 74.40% | 67.07% | 60.06% |
| Healthy vs. ILC | 85.28% | 81.61% | 89.16% | 89.5% | 77.06% | 76.52% |
| Healthy vs. DCIS | 80.34% | 80.27% | 73.99% | 67.65% | 81.94% | 78.83% |
| Healthy vs. PCR | 69.5% | 91.01% | 75.75% | 56.56% | 59.27% | 54.50% |
| Healthy vs. IDC+ILC | 75.26% | 76.27% | 80.48% | 79.70% | 70.29% | 70.42% |
| Healthy vs. IDC+ILC+DCIS | 73.25% | 75.70% | 78.81% | 79.22% | 72.53% | 69.3% |
| Mean AUC - all experiments | 74.80% | 78.94% | 78.06% | 74.5% | 71.36% | 68.25% |

**Table 4.** Table depicting performance metrics for both classifiers when comparing healthy tissue with invasive lobular cancer.

| Sample Quality | Accuracy (%) | Sensitivity (%) | Specificity (%) | AUC (%) | mAP (%) |
| --- | --- | --- | --- | --- | --- |
| All  (1 to 3) | 74.56 ± 9.67 | 75.87 ± 17.88 | 74.14 ± 16.48 | 82.13 ± 10.28 | 81.38 ± 9.39 |
| 3 | 80.18 ± 13.91 | 73.31 ± 19.91 | 86.08 ± 15.55 | 87.05 ± 12.09 | 83.85 ± 16.2 |

Table 5. Table depicting performance metrics for both classifiers when comparing healthy tissue with invasive ductal cancer.

| Sample Quality | Accuracy (%) | Sensitivity (%) | Specificity (%) | AUC (%) | mAP (%) |
| --- | --- | --- | --- | --- | --- |
| All  (1 to 3) | 76.11 ± 2.97 | 85.60 ± 1,67 | 56.73 ± 8.92 | 81.32 ± 3.78 | 89.3 ± 3.98 |
| 3 | 72.04 ± 11.44 | 61.06 ± 17.57 | 78.74 ± 17.15 | 78.38 ± 16.37 | 76.69 ± 16.6 |

Table 6. Table depicting performance metrics for both classifiers when comparing healthy tissue with ductal carcinoma in-situ.

| Sample Quality | Accuracy (%) | Sensitivity (%) | Specificity (%) | AUC (%) | mAP (%) |
| --- | --- | --- | --- | --- | --- |
| All  (1 to 3) | 83.48 ± 4.73 | 75.87 ± 17.88 | 90.54 ± 5.35 | 89.52 ± 7.44 | 82.60 ± 12.7 |
| 3 | 83.22 ± 10.73 | 55.72 ± 28.03 | 91.12 ± 12.52 | 87.62 ± 9.26 | 75.76 ± 16.5 |

Table 7. Table depicting performance metrics for both classifiers when comparing healthy tissue with tissue taken from patients who received NACT and achieved a pathological complete response.

| Sample Quality | Accuracy (%) | Sensitivity (%) | Specificity (%) | AUC (%) | mAP (%) |
| --- | --- | --- | --- | --- | --- |
| All  (1 to 3) | 88.85 ± 4.51 | 19.34 ± 21.81 | 96.26 ± 4.66 | 75.24 ± 16.32 | 34.66 ± 26 |
| 3 | 90.24 ± 3.85 | 35.29 ± 43.86 | 97.53 ± 1.84 | 88.15 ± 8.89 | 52.32 ± 29.3 |

**Confusion Matrices**


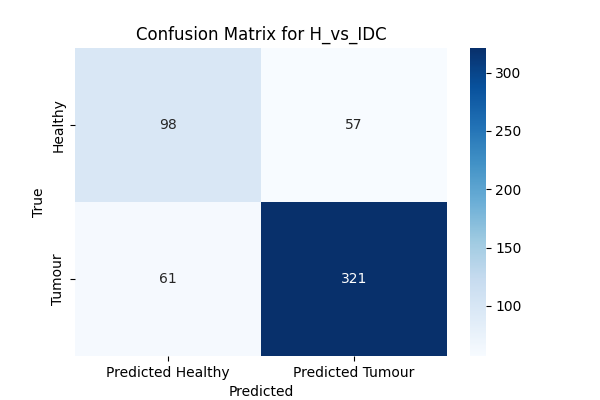


**Fig 1.** Confusion matrix comparing all tissue samples (quality 1 to 3) of healthy breast tissue versus invasive ductal carcinoma.


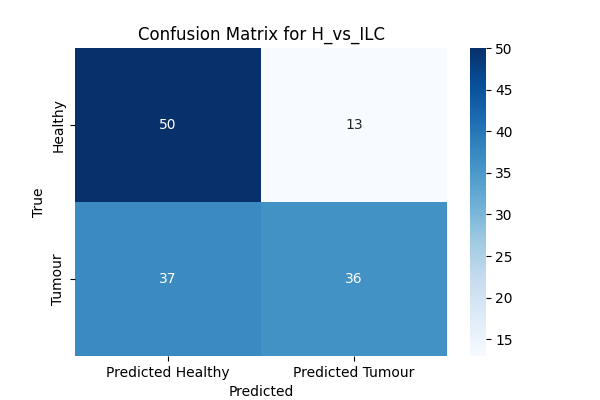


**Fig 2.** Confusion matrix comparing all tissue samples (quality 1 to 3) of healthy breast tissue versus invasive lobular carcinoma.


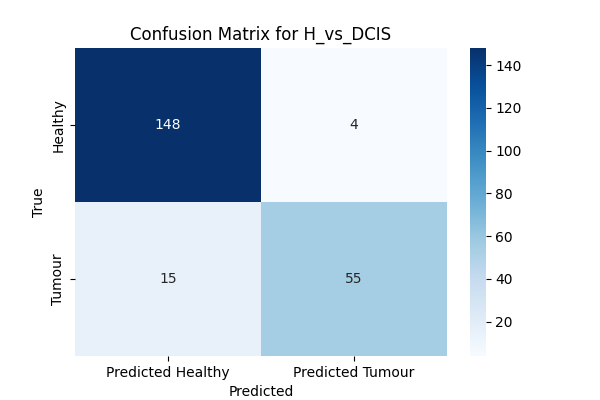


**Fig 3.** Confusion matrix comparing all tissue samples (quality 1 to 3) of healthy breast tissue versus ductal carcinoma in-situ.


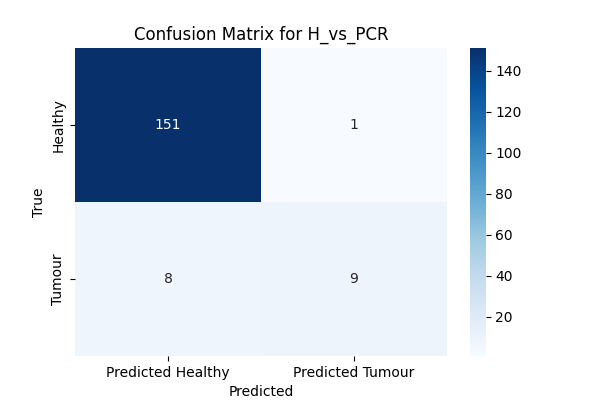


**Fig 4.** Confusion matrix comparing all tissue samples (quality 1 to 3) of healthy breast tissue versus pathological complete response (i.e. tissue that has received neoadjuvant chemotherapy).


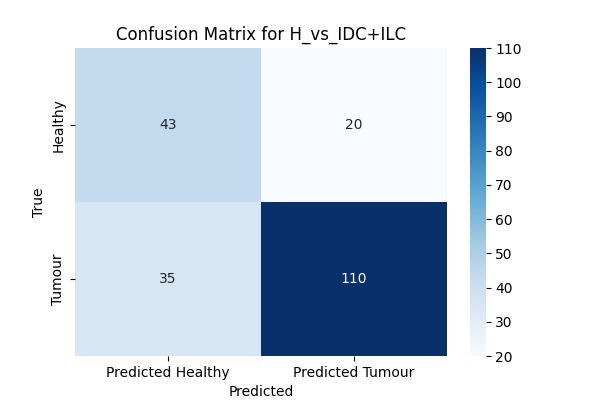


**Fig 5.** Confusion matrix comparing all tissue samples (quality 1 to 3) of healthy breast tissue versus invasive carcinoma types (invasive ductal and invasive lobular).


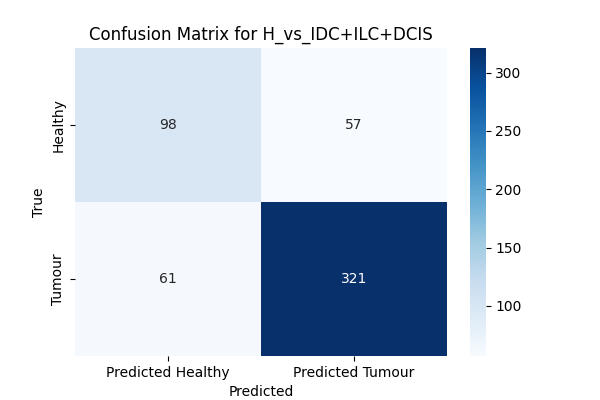


**Fig 6.** Confusion matrix comparing all tissue samples (quality 1 to 3) of healthy breast tissue versus pathological complete response (i.e. tissue that has received neoadjuvant chemotherapy).


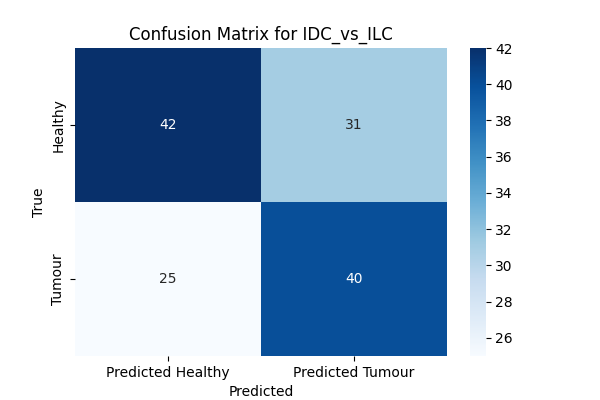


**Fig 7.** Confusion matrix comparing all tissue samples (quality 1 to 3) of invasive ductal carcinoma versus invasive lobular carcinoma.


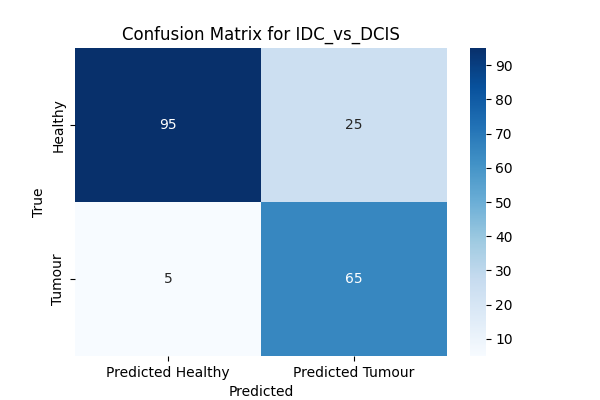


**Fig 8.** Confusion matrix comparing all tissue samples (quality 1 to 3) of invasive ductal carcinoma versus DCIS.


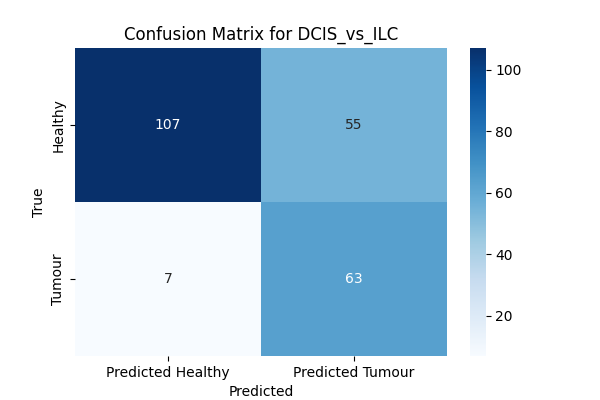


**Fig 9.** Confusion matrix comparing all tissue samples (quality 1 to 3) of DCIS versus invasive lobular carcinoma.
